# Supplementary figures and images for: Nuclear reassembly defects after mitosis trigger apoptotic and p53-dependent safeguard mechanisms in Drosophila
Source: PLoS Biol. 2024 Aug 26;22(8):e3002780. doi: 10.1371/journal.pbio.3002780 (PMC11379398; doi:10.1371/journal.pbio.3002780)

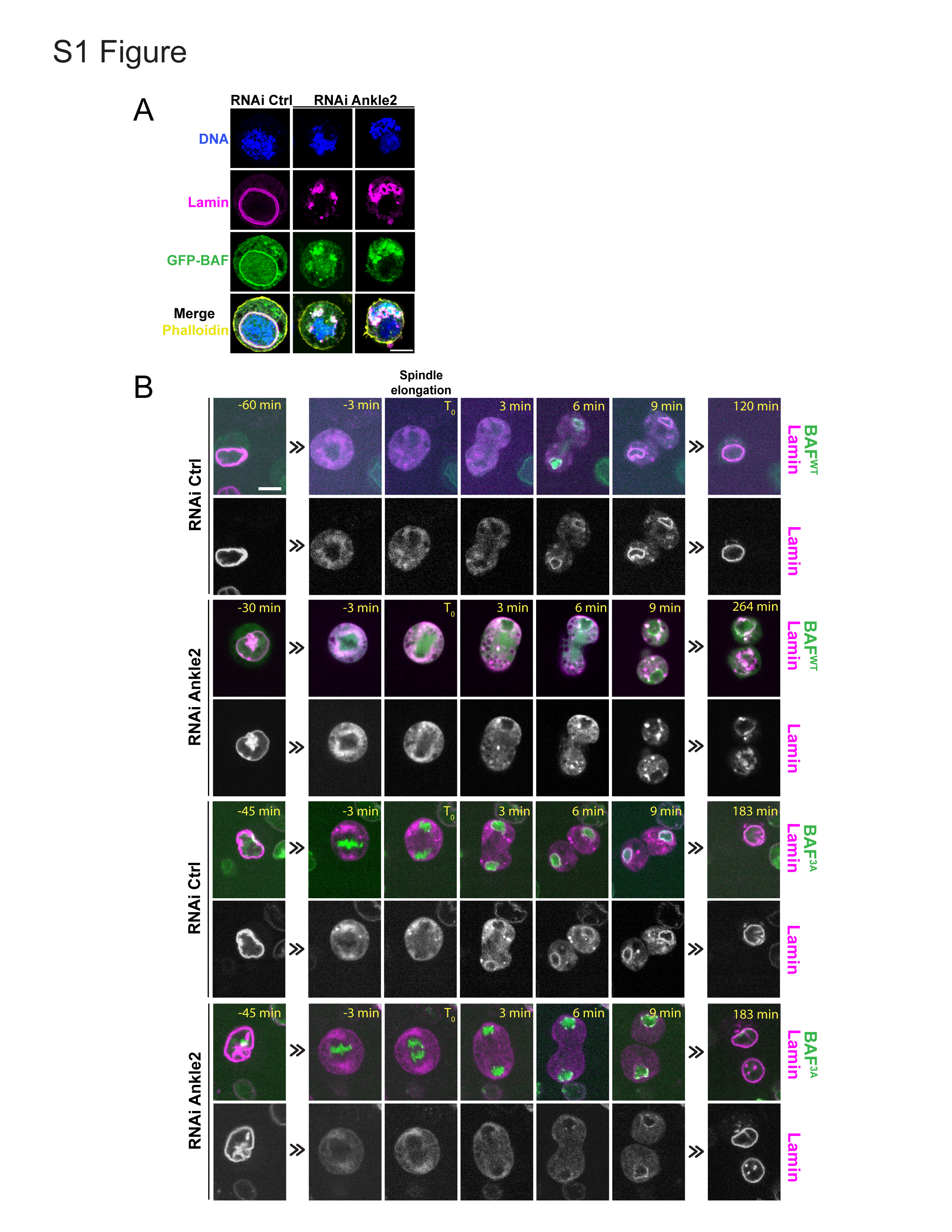

Supplement: S1 Fig — (A) Immunofluorescence showing the subcellular localization of GFP-BAF in D-Mel cells after RNAi depletion of Ankle2 and in control cells. Cells were stained for Lamin and with phalloidin to reveal actin. (B) Live imaging of mitosis in cells expressing GFP-BAFWT or GFP-BAF3A (green) and RFP-Lamin (magenta) after Ankle2 RNAi or Control RNAi. T0 was set as the beginning of spindle elongation in anaphase. Note that GFP-BAF3A stays on chromosomes throughout mitosis even when Ankle2 is depleted. In addition, the recruitment of RFP-Lamin at reassembling nuclei is rescued. Scale bars: 5 μm. (TIF) [file pbio.3002780.s001.tif]

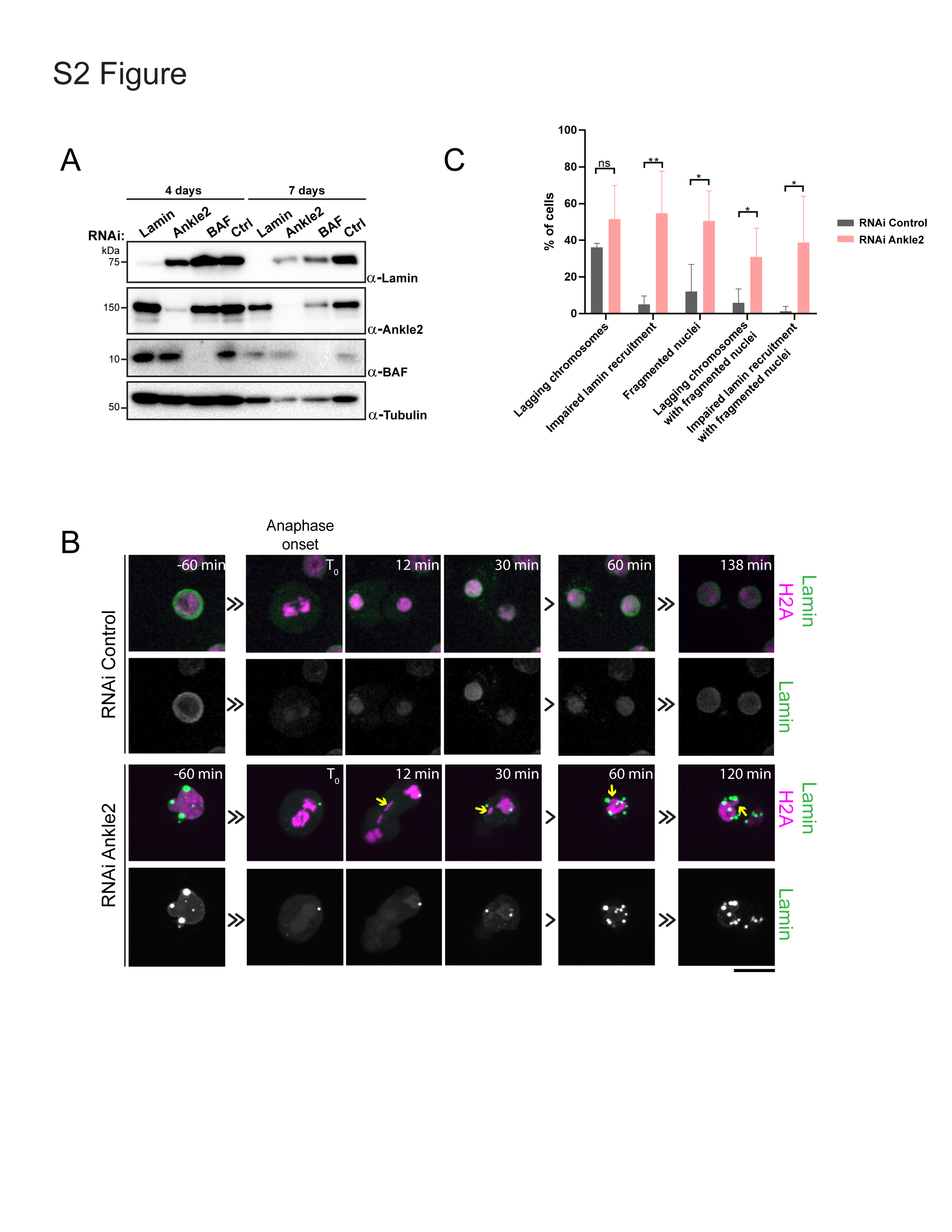

Supplement: S2 Fig — (A) Western blots showing RNAi depletion of Ankle2, BAF, and Lamin 4 or 7 days post-transfection. (B) Depletion of Ankle2 causes postmitotic fragmented nuclei. D-Mel cells expressing H2A-RFP and GFP-Lamin were RNAi-treated as indicated dsRNA for 4 days and mitoses were filmed. Arrows: a lagging chromosome in telophase becomes a micronucleus. Scale bar: 5 μm. (C) Quantification of nuclear defects observed by live imaging as in B. Averages of 4 experiments are shown, where 91 and 110 diving cells in total were scored for dsRNA control and dsRNA Ankle2, respectively. *p < 0.05, **p < 0.01, ns: nonsignificant from unpaired t tests. Coordinate values used to generate graph are available in S1 Data. (TIF) [file pbio.3002780.s002.tif]

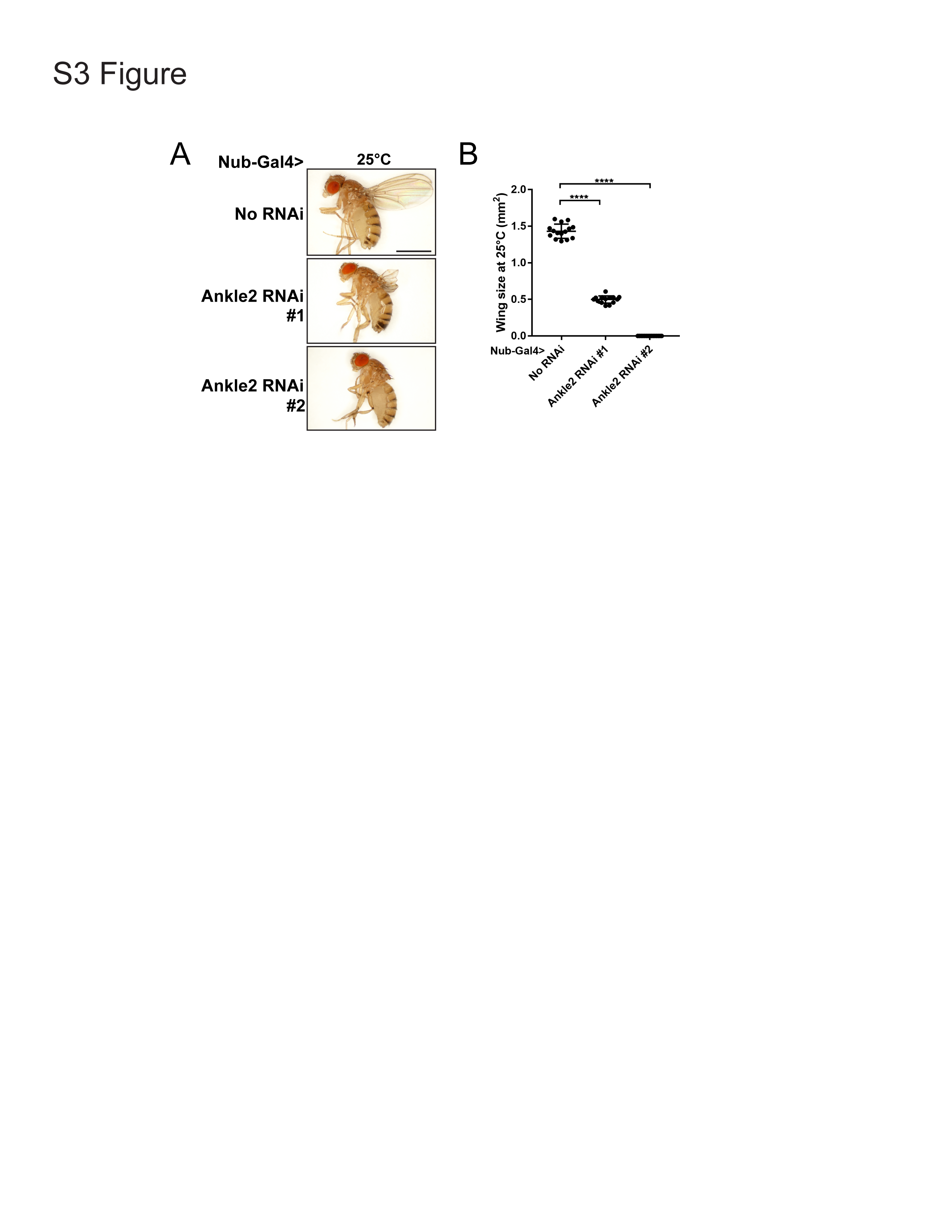

Supplement: S3 Fig — Ankle2 RNAi was driven by Nub-Gal4 at 25°C using lines VDRC100655 (Ankle2 RNAi line #1) and BDSC77437 (Ankle2 RNAi line #2). (A) Example images of adult flies. Scale bar: 1 mm. (B) Quantification of wing sizes at 25°C (n = 15). ****p < 0.0001 from unpaired t tests with Welch’s correction. Coordinate values used to generate graph are available in S1 Data. (TIF) [file pbio.3002780.s003.tif]

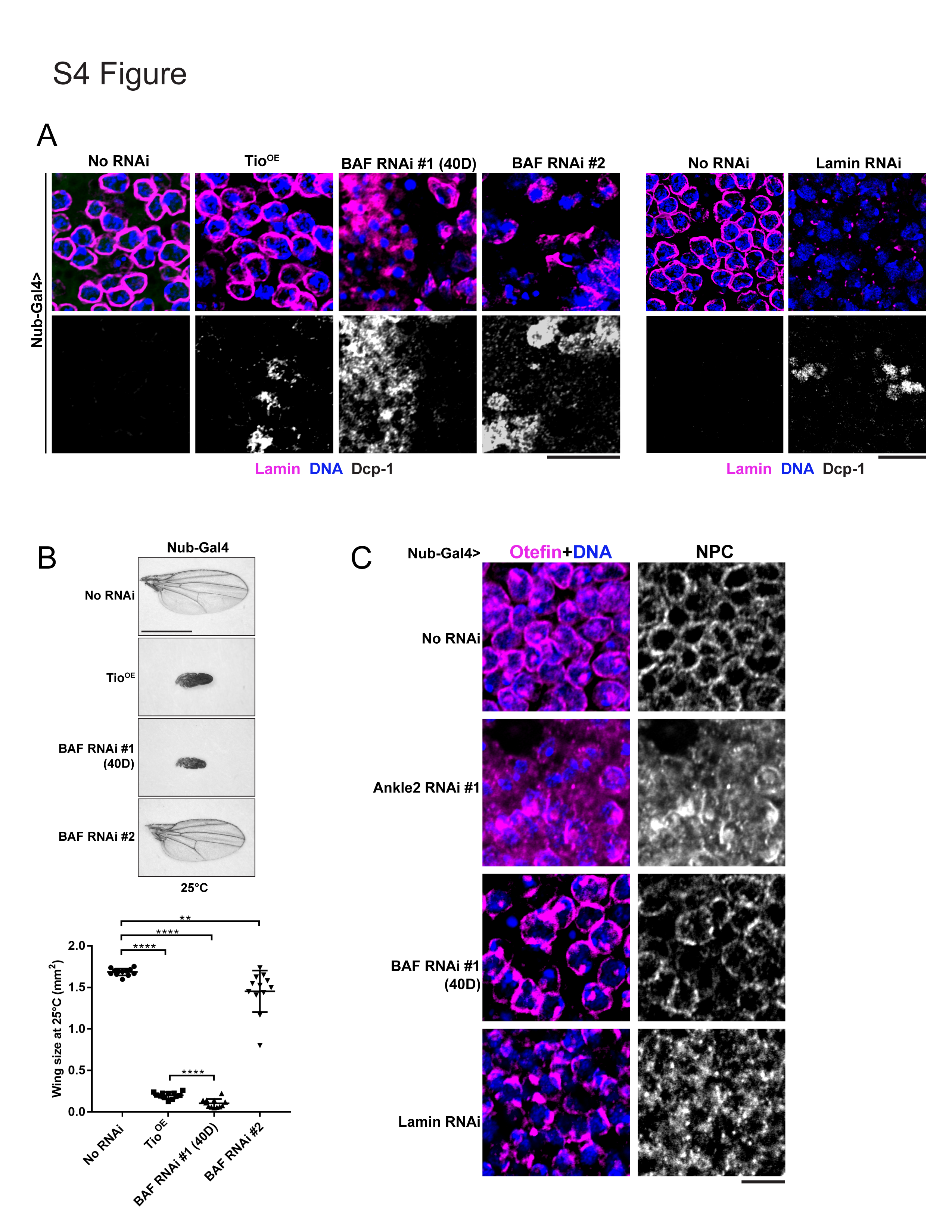

Supplement: S4 Fig — (A) Left: Nuclear defects in wing discs resulting from driving BAF RNAi from 2 lines (line #1: VDRC103013; line #2: BDSC36108) with Nub-Gal4 at 25°C. Both BAF RNAi constructions induce Lamin mislocalization and DNA foci devoid of Lamin. Line #1 (VDRC103013) has the BAF RNAi construction inserted at cytolocation 40D which was shown to also lead to overexpression of Tio. A control line with a UAS element alone inserted at the same site (VDRC60101, TioOE) does not result in similar nuclear defects. Therefore, these defects are specific to BAF depletion for both BAF RNAi lines. However, the TioOE line results in some level of apoptosis revealed by Dcp-1 staining. Right: Lamin RNAi also induces apoptosis as indicated by Dcp-1 staining. Results from separate experiments are shown in the left and right parts. Scale bar: 10 μm. (B) Adult wing defects resulting from driving the 2 BAF RNAi insertions with Nub-Gal4 at 25°C. Top: Examples of adult wings of the indicated genotypes. Line #1 (VDRC103013) results in a more pronounced phenotype but the control TioOE line results in a similar small wing phenotype. Therefore, the adult wing phenotype with line #1 is not specific to BAF depletion. Scale bar: 1 mm. Bottom: Quantification of wing sizes at 25°C (n = 12). **p < 0.01, ****p < 0.0001, ns: nonsignificant from unpaired t tests with Welch’s correction. (C) RNAi Depletion of Ankle2 (line #1, VDRC100655), BAF (line #1, VDRC103013) or Lamin results in mislocalization of Otefin and NPC proteins. All constructions were driven with Nub-Gal4 at 25°C and wings discs were analyzed by immunofluorescence. Scale bar: 5 μm. Coordinate values used to generate graph are available in S1 Data. (TIF) [file pbio.3002780.s004.tif]

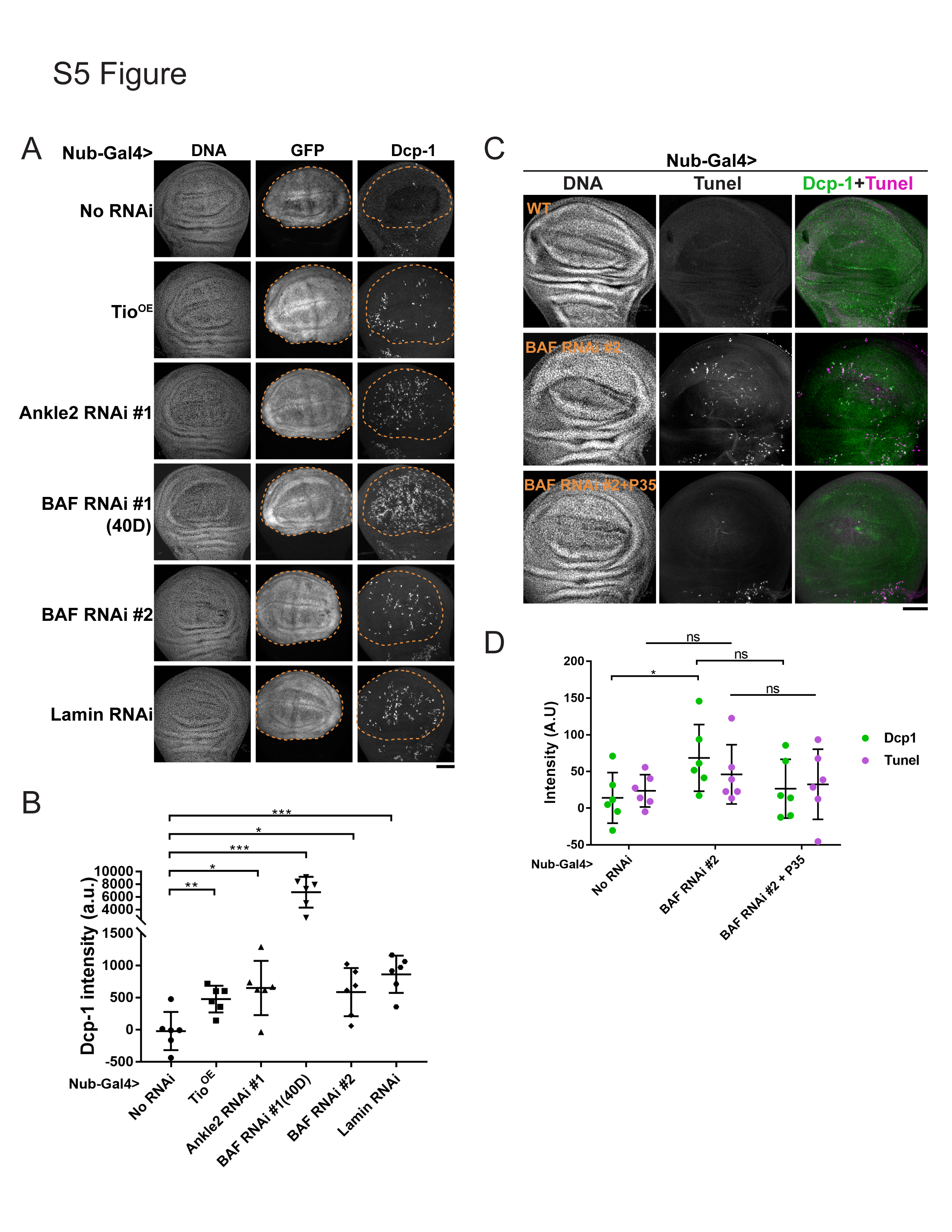

Supplement: S5 Fig — (A) The indicated RNAi constructions were induced in the wing pouch by Nub-Gal4 at 25°C. In parallel, UAS-GFP was used as a marker of the region of interest (wing pouch, dotted lines). Wing discs were analyzed by immunofluorescence against cleaved Dcp-1 and stained for DNA (DAPI). (B) Quantification of the Dcp-1 intensities measured in the wing pouch region of individual wing discs of the indicated genotypes as in A (n = 6). (C) Detection of apoptosis by TUNEL (magenta) and simultaneous immunofluorescence for cleaved Dcp-1 (green) in wing discs depleted of BAF. Expression of P35 abrogates apoptosis. (D) Quantifications of TUNEL and Dcp-1 signals in wing discs of the indicated genotypes (n = 6). In all panels: BAF RNAi #1: VDRC103013; BAF RNAi #2: BDSC36108; Ankle2 RNAi #1: VDRC100655. All Scale bars: 50 μm. All error bars: SD *p < 0.05, **p < 0.01, *** p < 0.001, ns: nonsignificant from unpaired t tests with Welch’s correction. Coordinate values used to generate graphs are available in S1 Data. (TIF) [file pbio.3002780.s005.tif]

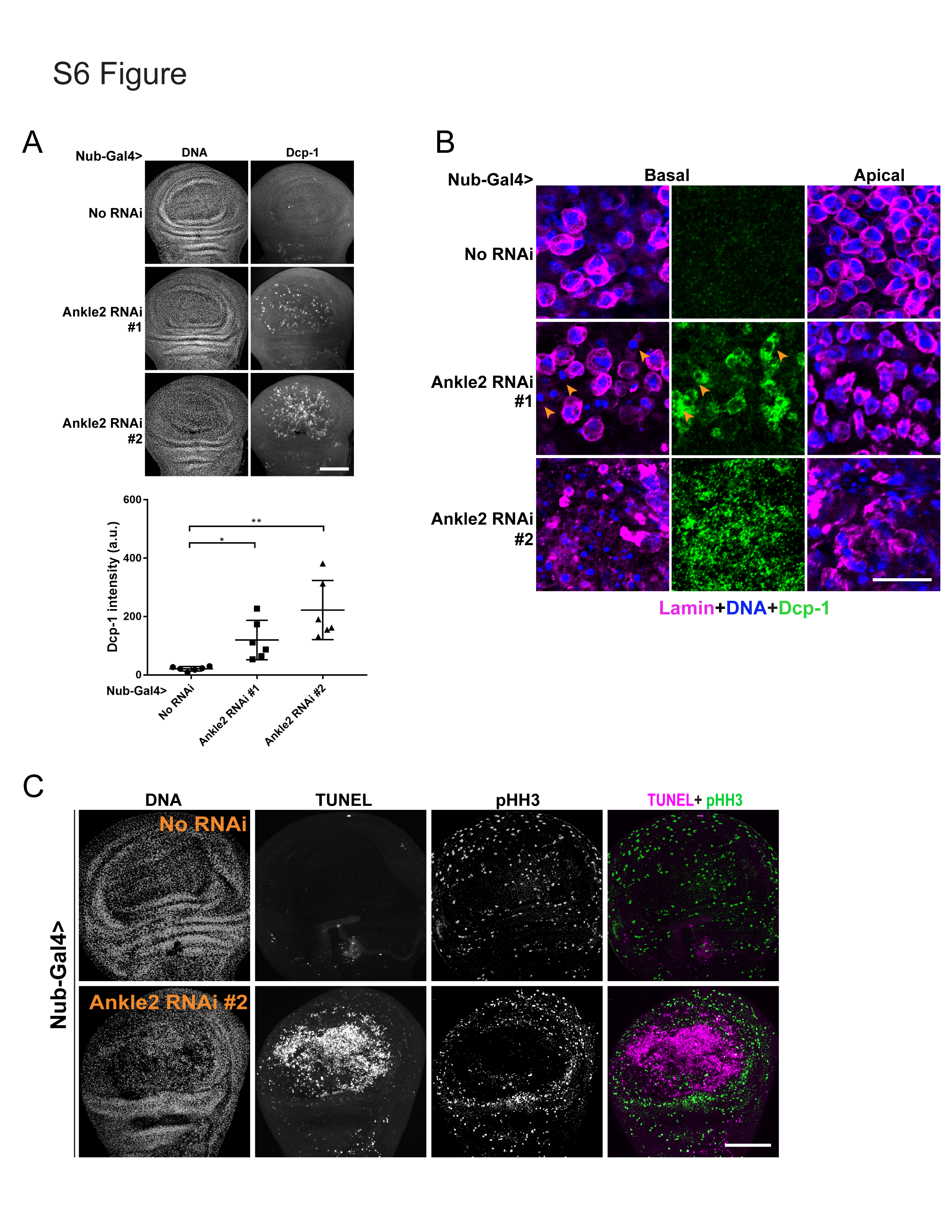

Supplement: S6 Fig — (A) Ankle2 RNAi was driven by Nub-Gal4 at 25°C using lines VDRC100655 (Ankle2 RNAi line #1) and BDSC77437 (Ankle2 RNAi line #2). Top: Cleaved Dcp-1 staining in wing discs of the indicated genotypes. Bottom: Quantification of Dcp-1 signals (n = 6). All error bars: SD *p < 0.05, **p < 0.01, from unpaired t tests with Welch’s correction. (B) Nuclear defects and apoptosis are revealed by immunofluorescence against Lamin, DAPI, and cleaved Dcp-1. Arrowheads: hypercondensed DNA in apoptotic cells. Scale bar: 10 μm. (C) phospho-Histone H3 (pHH3) staining suggests compensatory cell proliferation around the area of the wing disc where Ankle2 is depleted (line #2, BDSC77437) and apoptosis occurs (detected by TUNEL). All scale bars for imaginal wing discs: 50 μm. Coordinate values used to generate graph are available in S1 Data. (TIF) [file pbio.3002780.s006.tif]

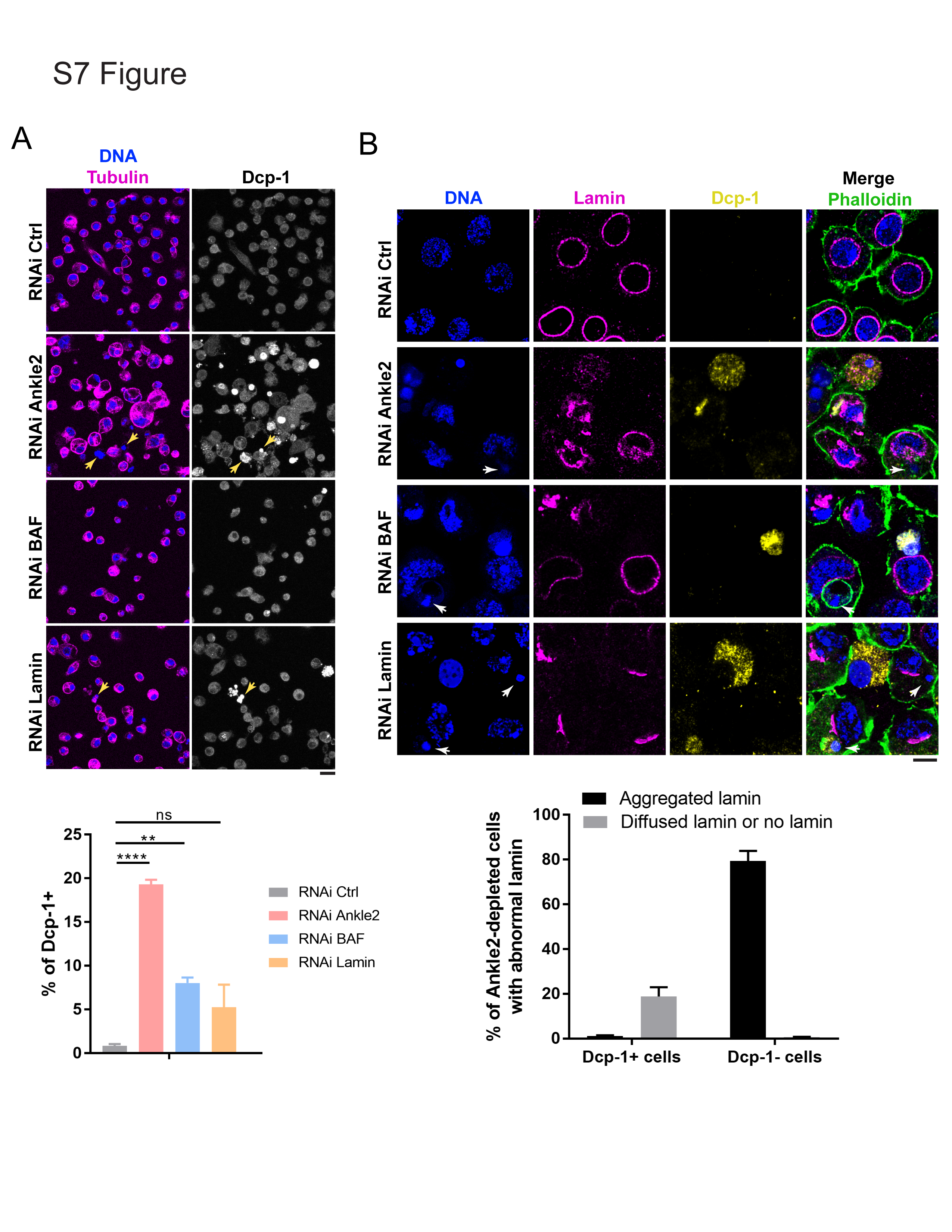

Supplement: S7 Fig — (A) D-Mel cells were transfected with the indicated dsRNA and analyzed by immunofluorescence after 4 days. Top: Examples of images. Arrows: Apoptotic cells with hypercondensed DNA. Scale bar: 20 μm. Bottom: Quantification of Dcp-1 positive (Dcp-1+) cells of the indicated conditions. Averages of 3 experiments are shown, where 292 to 395 cells were scored per experiment in each condition. (B) Top: Apoptosis is observed in a fraction of nuclear-defective cells. D-Mel cells were RNAi-treated as indicated and analyzed by immunofluorescence after 4 days. Arrows: Examples of cells with nuclear defects that are Dcp-1-negative. Bottom: Ankle2-depleted cells with Lamin defects were scored for Dcp-1 staining. Averages of 3 experiments are shown, where 256 to 333 cells were scored per condition in each experiment. All error bars: SD **p < 0.01, ****p < 0.0001, ns: nonsignificant from paired t tests. Coordinate values used to generate graphs are available in S1 Data. (TIF) [file pbio.3002780.s007.tif]

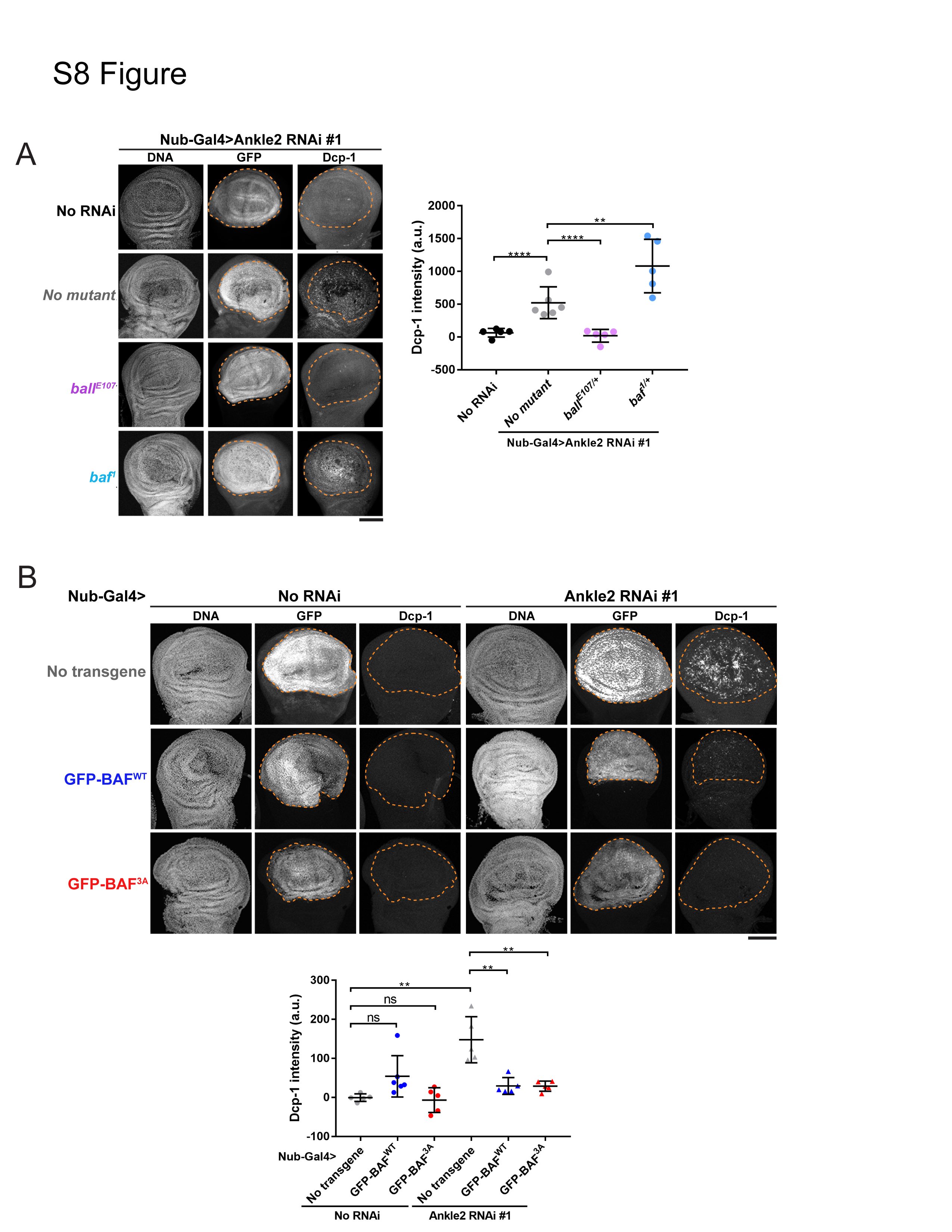

Supplement: S8 Fig — (A) Mutation in baf enhances, while mutation in ball suppresses, apoptosis resulting from Ankle2 depletion. Left: Examples of wing discs of the indicated genotypes after induction of Ankle2 RNAi using Nub-Gal4 at 25°C. In parallel, UAS-GFP was used as a marker of the region of interest (wing pouch, inside dotted line). Right: Quantification of Dcp-1 signals in the wing pouch (n = 5 to 6). (B) Overexpression of GFP-BAF3A or GFP-BAFWT rescues apoptosis resulting from Ankle2 depletion. Top: Examples of wing discs of the indicated genotypes at 25°C. Bottom: Quantification of Dcp-1 signals (n = 5 to 6). Analysis was done as in A. In all experiments, Ankle2 depletion was done using line #1 (VDRC100655). All scale bars: 50 μm. All error bars: SD **p < 0.01, ****p < 0.0001, from unpaired t tests with Welch’s correction. Coordinate values used to generate graphs are available in S1 Data. (TIF) [file pbio.3002780.s008.tif]

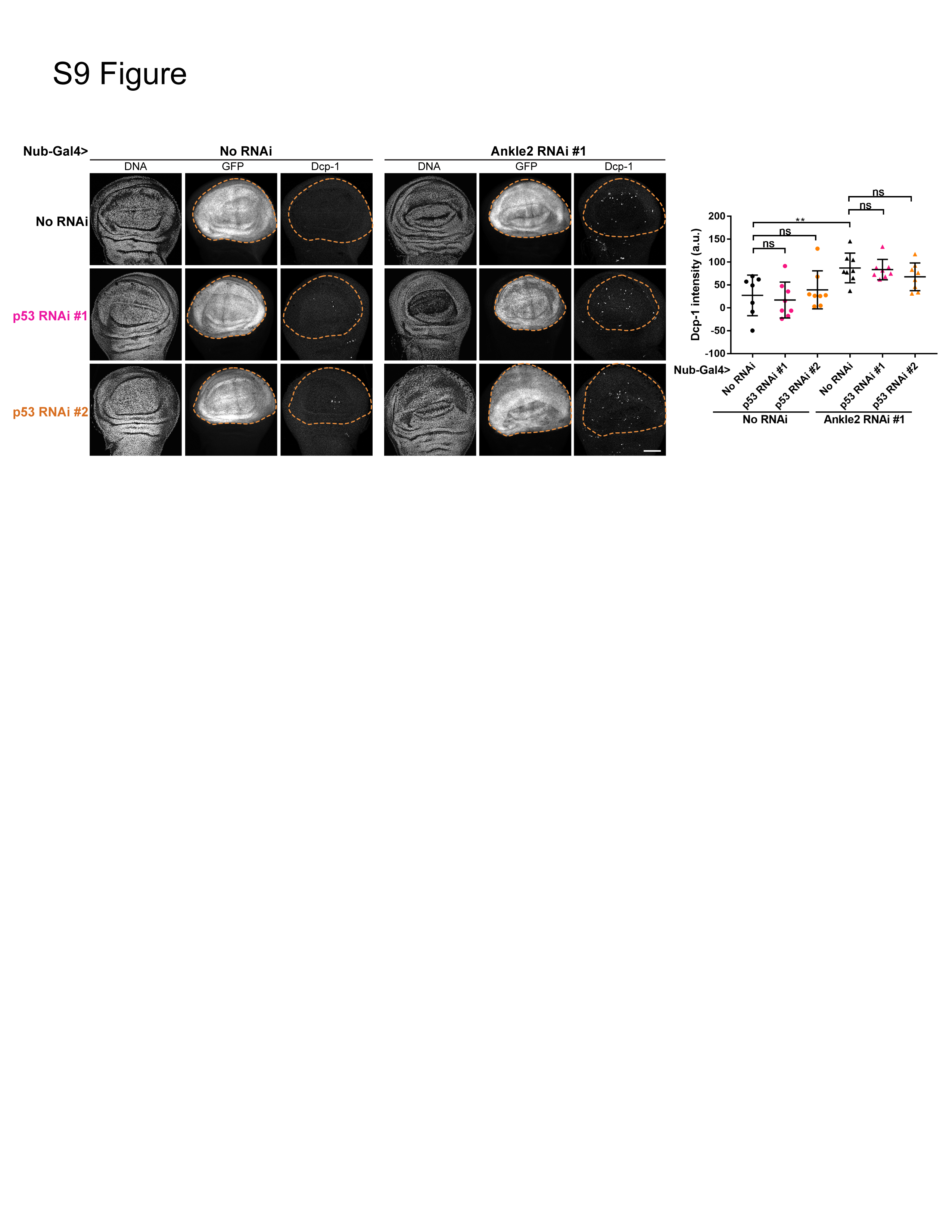

Supplement: S9 Fig — (A) Depletion of p53 does not block apoptosis resulting from Ankle2 depletion (line VDRC100655). RNAi depletions of p53 were achieved with lines VDRC38235 (p53 RNAi #1) and VDRC10692 (p53 RNAi #2). Left: Examples of wing discs of the indicated genotypes after induction of Ankle2 RNAi using Nub-Gal4 at 25°C, UAS-GFP is used as a marker for the region of interest (pouch area, inside dotted line). Right: Quantifications of Dcp-1 signals in the wing pouch (n = 7 to 8). Scale bar: 50 μm. All error bars: SD **p < 0.01, ns: nonsignificant from unpaired t tests with Welch’s correction. Coordinate values used to generate graph are available in S1 Data. (TIF) [file pbio.3002780.s009.tif]

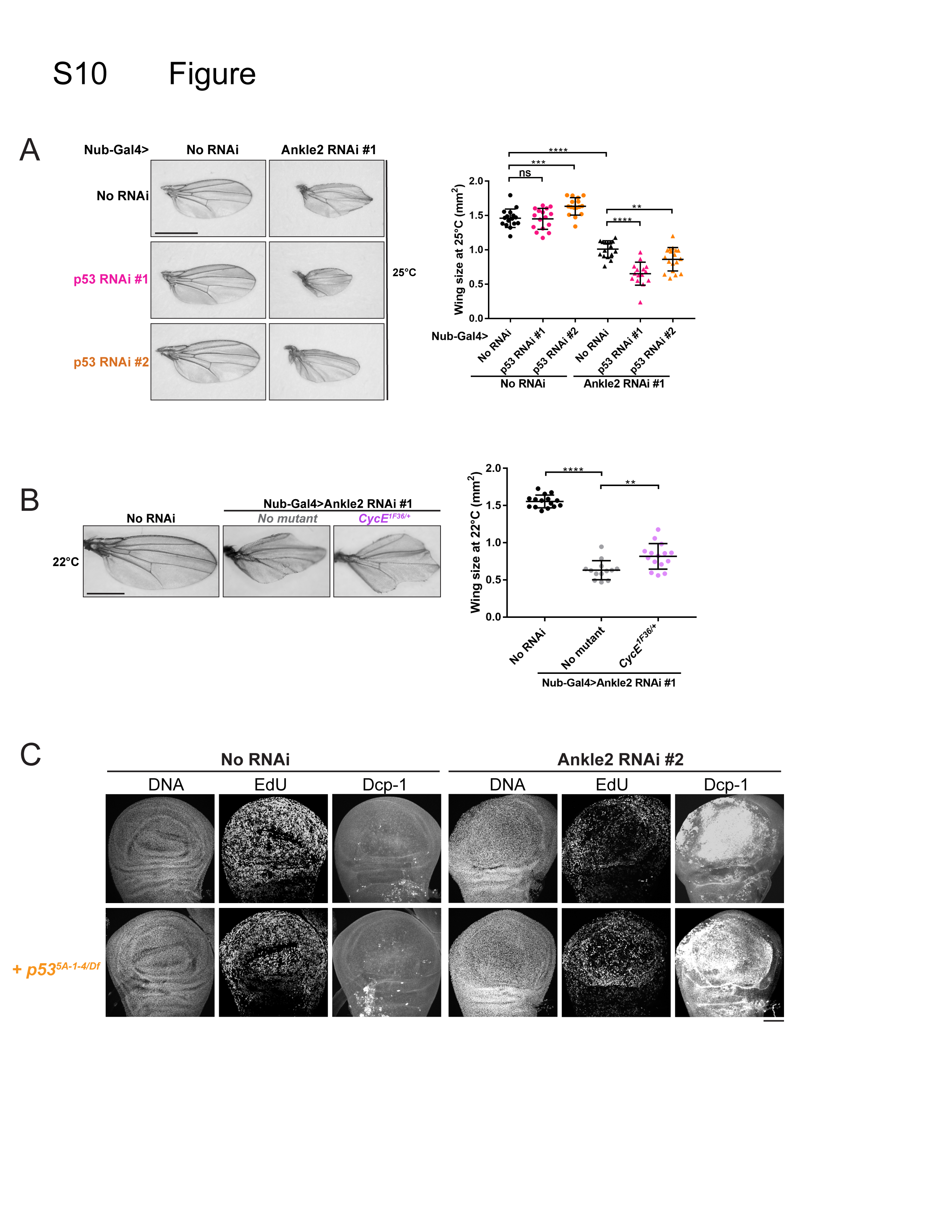

Supplement: S10 Fig — (A) Depletions of p53 enhance the small wing phenotype resulting from Ankle2 depletion. Left: Examples of adult wings of the indicated genotypes after inducing RNAi using Nub-Gal4 at 25°C. Right: Quantifications of wing sizes at 25°C (n = 15 to 18). Scale bar: 1 mm. (B) Mutation in cycE enhance the small wing phenotype resulting from Ankle2 depletion. Left: Examples of adult wings of the indicated genotypes at 22°C. Right: Quantifications of wing sizes (n = 13 to 15). Scale bar: 1 mm. In panels A and B, Ankle2 RNAi line #1 (VDRC100655) was used. All error bars: SD **p < 0.01, ***p < 0.001, ****p < 0.0001, ns: nonsignificant from unpaired t tests with Welch’s correction. (C) A decrease in the population of EdU+ cells resulting from Ankle2 depletion (line #2, BDSC77437) in wing discs is rescued by inactivation of p53. Wings discs of indicated genotypes were incubated with EdU for 1 h and then co-stained for DNA (DAPI) and cleaved Dcp-1. Scale bar: 50 μm. Coordinate values used to generate graphs are available in S1 Data. (TIF) [file pbio.3002780.s010.tif]
