# Supplementary material for: Nuclear reassembly defects after mitosis trigger apoptotic and p53-dependent safeguard mechanisms in Drosophila
Source: PLoS Biol. 2024 Aug 26;22(8):e3002780. doi: 10.1371/journal.pbio.3002780 (PMC11379398; doi:10.1371/journal.pbio.3002780)
Supplement: S1 Raw Images — (PDF) [file pbio.3002780.s017.pdf]

Raw images of Western-blots in Fig. 1A

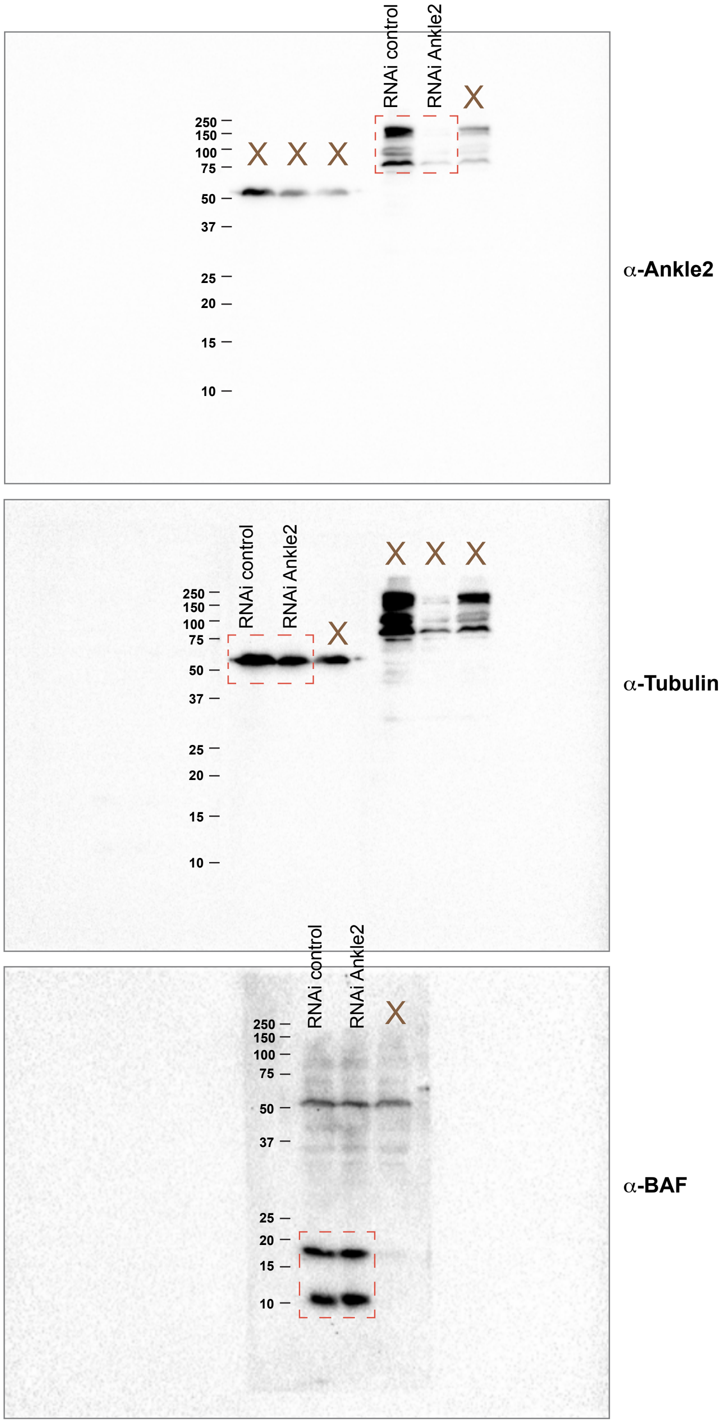

Western blots were revealed using the Clarity Western ECL substrate kit.
Images were taken using a ChemiDoc system.
Cropped images shown in Fig 1A are indicated by red rectangles.

Raw images of Western-blots in Fig. 1H

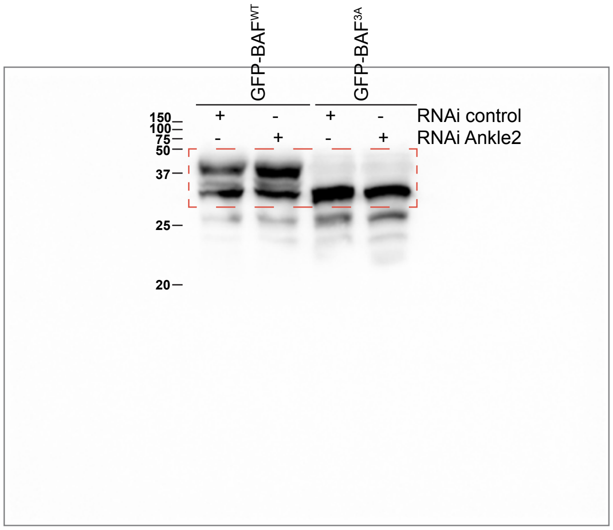

α-GFP

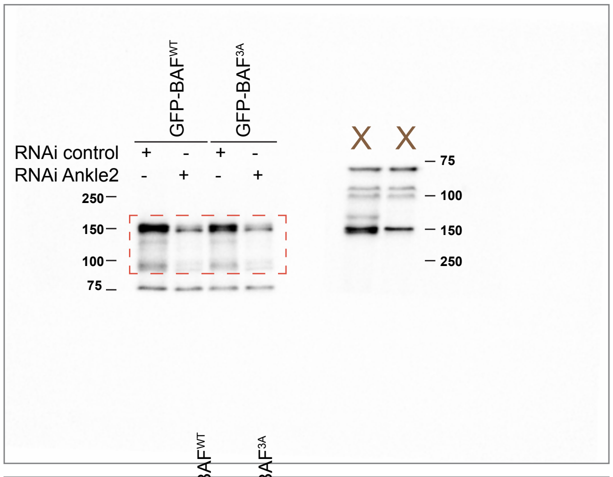

α-Ankle2

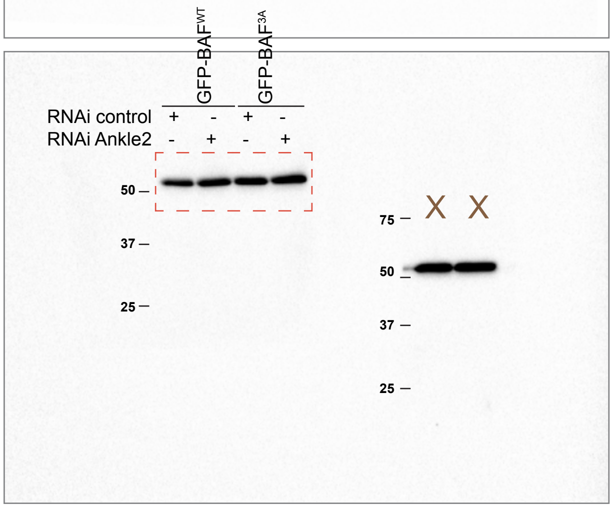

α-Tubulin

Western blots were revealed using the Clarity Western ECL substrate kit.
Images were taken using a ChemiDoc system.
Cropped images shown in Fig 1H are indicated by red rectangles.

Raw images of Western-blots in Fig. S2A

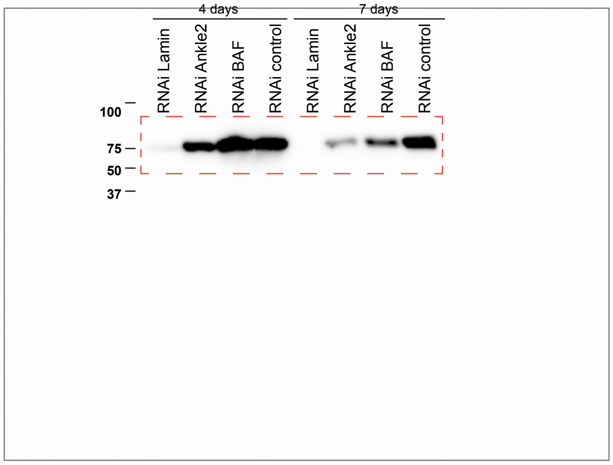

α-Lamin

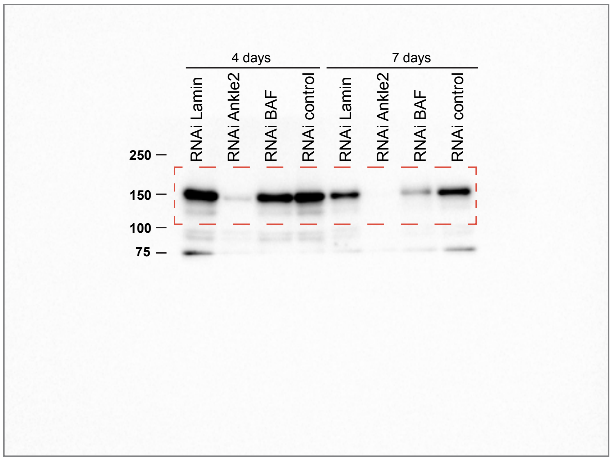

α-Ankle2

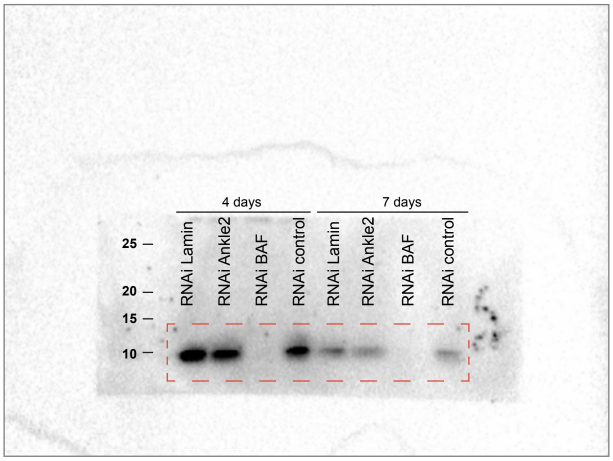

α-BAF

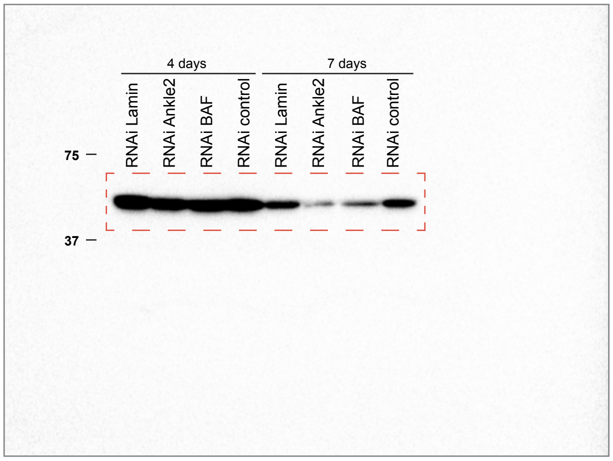

α-Tubulin

Western blots were revealed using the Clarity Western ECL substrate kit.
Images were taken using a ChemiDoc system.
Cropped images shown in Fig S2A are indicated by red rectangles.
