# Supplementary material for: Nuclear reassembly defects after mitosis trigger apoptotic and p53-dependent safeguard mechanisms in Drosophila
Source: PLoS Biol. 2024 Aug 26;22(8):e3002780. doi: 10.1371/journal.pbio.3002780 (PMC11379398; doi:10.1371/journal.pbio.3002780)
Supplement: Attachment — Submitted filename: Response to reviewers comments.pdf [file pbio.3002780.s018.pdf]

To: Dr Ines Alvarez-Garcia

Editor, PLoS Biology

Dear Dr Alvarez-Garcia,

We thank the reviewers for their thoughtful comments and suggestions. We were very pleased to read that our manuscript was well received. Please find our responses in blue.

Reviewers' comments

Rev. 1:
BAF localizes on chromosomes at mitotic exit. This is due to BAF dephosphorylation by PP2A and Ankle2 and is essential for appropriate nuclear envelope reformation. This manuscript from Li et al nicely recapitulates the relationship between Ankle2, BAF and lamin that was described in other systems, and their role in post-mitotic nuclear envelope assembly and tissue integrity in Drosophila. The authors provide an analysis of the consequences of perturbing this process in vivo, in Drosophila imaginal discs. Wing discs with defective nuclear envelopes accumulate dying cells, and lead to small-winged adults. Similar to previous work, they find that lamina defects induce DNA damage, which in turn trigger apoptosis by activation of Chk2. Surprisingly, inhibition of apoptosis does not ameliorate - but exacerbates - the adult phenotype, suggesting that apoptosis promotes tissue integrity. In addition, the authors find that p53-mediated cell cycle arrest also contributes to tissue integrity. This manuscript is clear and well written, and the authors are fair in their claims and cite relevant previous work. Within its scope, this manuscript is interesting and brings a new perspective on nuclear integrity and how this affects epithelial tissue homeostasis. However, there are some major points that I believe should be addressed before accepting this manuscript for publication.

1) Developmental defects in the wings and wing discs were reported for dMAN1 mutant flies (see PMID 18723885). Similarly, lamin depletion using ap-Gal4 causes cell death and defects in adult wings (see PMID 20505245). Unless I missed them, these works should be cited.

We have now cited these works.

2) The authors suggest several times that p53-mediated cell cycle arrest improves cell viability by allowing time for nuclear repair to occur. This is something that could be easily tested. Does depletion of CycE reduce the levels of DNA damage induced by Ankle2 RNAi?

We agree that this is a reasonable prediction based on the model we propose. We have taken the time to conduct this experiment. The genetic crosses required took several generations. First, we obtained two CycE RNAi insertions on the 3rd chromosome that we had to double-balance. We could then combine these transgenes with the Ankle2 RNAi (2nd chromosome, also double-balanced) in the same line, and then cross this line with the Nub-Gal4 line. The results we obtained are shown below (combined results from 3 experiments, 22 wing discs per condition in total). We did observe a reduction of DNA damage (pH2Av signal) upon depletion of Ankle2 with both CycE RNAi insertions. However, a partial rescue was also obtained with a mCherry RNAi non-target

control. This result suggests that the introduction of a second UAS (UAS-Ankle2 RNAi + UAS-CycE RNAi or UAS-cCherry RNAi) causes a dilution of Gal4 that weakens Ankle2 RNAi and the associated DNA damage. Nevertheless, the rescue is significantly stronger with CycE RNAi insertion #1 than with mCherry RNAi, suggesting that the depletion of CycE is responsible for some level of rescue. While these results are consistent with our model, the differences observed are small. We are therefore reluctant to draw firm conclusions from these results, and prefer not to include them in our manuscript.

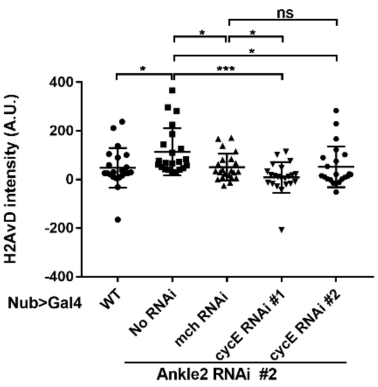

Interestingly, despite a possible Gal4 dilution effect as above, the pH2Av signal is increased when apoptosis is blocked by expression of UAS-P35 (Fig 6A), reinforcing the idea that apoptosis may be triggered in cells incurring DNA damage.

3) As visible from Figure 2A, the scoring of micronuclei should be revised, and the quantifications updated accordingly. The micronuclei highlighted by yellow arrows in the panels showing Ankle2 and BAF RNAi are as large as main nuclei - and, therefore, can't be called micronuclei. Micronuclei are normally small, much smaller than the main nucleus, they may contain one or few mis-segregated chromosomes. They are normally visible as dense DAPI spots, and although there is not a defined size, I believe the scoring used by the authors is not appropriate.

We understand this concern. We have now changed the name of the “micronuclei” category for the more conservative term “fragmented nuclei” in Fig 2C. Compared to vertebrate cells, micronuclei containing one or a subset of chromosomes could be relatively quite large, given that *Drosophila* has only 4 chromosomes. Nevertheless, from our images, we cannot be sure that apparent separate DNA masses are genuine micronuclei. We have also changed the names of scored categories in Fig S2C.

4) DNA damage from abnormal NE causes Chk2-dependent, but p53-independent apoptosis in wing discs where Ankl2 has been knocked down. This suggests than non-apoptotic cell death could be eliminating those cells that are retained as a result of p35 overexpression. These results are interesting, especially in light of what happens when DNA damage is caused by caspase activation. I believe they should be discussed and contextualized with the work of the Milan lab (PMID 37751744).

These are insightful ideas. We have added discussion points about potential contributions of non-apoptotic cell death and the induction of cell migration by caspases and DNA damage in view of the work by the Milan lab.

5) I disagree with the authors' perspective on undead cells in the discussion. The retention of undead cells should not prevent compensatory proliferation. If anything, it should enhance it, because cells with active caspase are retained in the tissue and therefore have the ability of continuing producing growth signals (the authors mention this themselves later). From the results presented, it is clear that retention of abnormal cells is deleterious, because blocking apoptosis,

while inhibiting cell death in the disc, is insufficient to rescue the small wing phenotype elicited by abnormal NE in the adult. This could be due to the occurrence of non-apoptotic cell death at later stages of development. Non apoptotic cell death could eliminate those cells that are retained as a result of p35 overexpression. While still in the disc, these undead cells could also exacerbate the phenotype by inducing non-autonomous cell death. These possibilities should be discussed.

We have re-organized our discussion of these points, which needed to be clarified. We actually agree with the reviewer regarding undead cells and compensatory proliferation. We have added discussion points regarding non-apoptotic damaged cells (as mentioned in response to the previous point).

6) I suggest reviewing the introduction. Especially the section on micronuclei seems out of place, interrupting an otherwise linear flow of thoughts on nuclear integrity.

We have re-worked the introduction in response to the reviewer's suggestions. The paragraph about micronuclei has been moved. See also our response to specific suggestions about the Introduction below.

Minor comments:

Figures:

Figure 1A, WB: why are there multiple bands for Ankle2? Although the RNAi diminishes the signal from all bands, suggesting that the antibody does indeed recognize Ankle2, it would be reassuring to test the antibody in a mutant or in an overexpression. If mutations are lethal, maybe in flip-out clones with immunofluorescence (if the antibody works in immunofluorescence?)

We have repeatedly observed multiple bands for Ankle2 that are sensitive to Ankle2 RNAi. They likely originate from splice variants of Ankle2 which is encoded from 9 exons. Our result is sufficient to establish the specificity of our antibody in Western blot and to verify the depletion of Ankle2. We do not think it is necessary to test the antibody in mutant flies. We also note that the antibody did not work conclusively in immunofluorescence.

Figure 1B: [Assuming the labeling is incorrect, and RNAi NT is actually the left panel] Is Flag-BAF enrichment at the NE lost because the NE is compromised, or because BAF is not correctly targeted? It would be helpful to see a staining for lamin. In addition, the cell shown has condensed and fragmented DNA. Can the authors stain with antibodies against cDcp1 to demonstrate that the cell is not dead, or show a cell with less nuclear alterations?

We have corrected the labeling in Fig 1B; thank you. We have now conducted additional immunofluorescence experiments as proposed. We have stained GFP-BAF cells for DNA, Lamin and actin (phalloidin). We found that the loss of GFP-BAF at the nuclear periphery (edges of the DNA) co-occurs with a mis-localization of Lamin. This data is in the new Figure S1A. These observations are consistent with our observation that BAF recruitment to DNA is required for the correct recruitment of Lamin at reassembling nuclei (Fig 2). As requested by the reviewer, we have

also stained cells depleted by Ankle2, BAF or Lamin with antibodies against Dcp-1 (Fig S7). In each case, we find cells with aberrant DNA and Lamin organization that are Dcp-1 negative (New Fig S7B top, white arrows). Among the Ankle2-depleted cells with defective Lamin, the majority are Dcp-1 negative (New Fig S7B bottom). These results are consistent with our results in wing discs, where we have shown that blocking apoptosis does not prevent nuclear defects (Fig 5A), suggesting that the observed nuclear defects are not a mere consequence of apoptosis.

Figure 2A: I think it would be important to differentiate which NE defects are due to Ankle2/BAF/lamin depletion and which defects are a consequence of apoptotic cellular dismantling (partially answered in Figure 5A). Could the authors discriminate between cells that are dead and cells that are alive (maybe with a cDcp1 staining)? Also, some of the cells shown appear much larger than controls. Are defects in cell division occurring in these cells as well? This seems to be the case, given the quantifications on multinucleation (Fig. 2C, although the % reported in the quantification does not match the frequency that can be inferred looking at Fig. 2A). Note that DNA damage could cause defects in cell division, and defects in cell division might exacerbate the observed defects in the NE

Regarding the first question, please see our answer to the preceding point and refer to the new Fig S7B.
Regarding the second question, the D-Mel cell line used shows intrinsic heterogeneity in cell size. We do see defects in large cells as well as in small cells (see arrows pointing at defects in the larger cells in Fig 2A). We did not detect an obvious increase in cell division or chromosome segregation defects (Fig S2C) after depletion of Ankle2. However, we agree that the modest increase in bi/multinucleation we observed (Fig 2C) suggests an occurrence of cell division defects that could be indirect consequences of nuclear reassembly defects and DNA damage in the previous cell cycle.

Figure 3B: Why do the authors think that lamin depletion has such a small effect on adult wing size compared to Ankle2?

We have discussed this interesting point in our Discussion section. In brief, we suggest that this difference reflects the Lamin independent part of Ankle2-BAF functions. See this text:
"…interfering with BAF function in Drosophila results in a failure to recruit Lamin, consistent with the fact that BAF forms a complex with Lamin in Drosophila (43, 78). However, we found that while depletion of Lamin leads to NR defects, apoptosis and developmental defects, these phenotypes tend to be even stronger when Ankle2 or BAF is depleted. This suggests that the known role of BAF in cross-bridging chromosomes in telophase to ensure the formation of a single nucleus of normal shape (independently of lamins) is a crucial, conserved aspect of its functions in NR (7)."

S1A: the data relative to 7 days are very difficult to interpret due to the variability in the loading control (is this due to high level of cell death? Off target of the RNAi? Or just insufficient loading?)

The differences in Tubulin signal after 7 days are due to decreased proliferation and increased cell death when Ankle2 or BAF is depleted. For 7 days of RNAi, cells were diluted, re-plated and re-

transfected after 4 days. After only 4 days of RNAi, the depletion of the protein is too recent to result in marked differences in cell numbers.

S1B: although I do clearly see an acentric chromosome that will likely reside in a micronucleus, I do not see the micronucleus at 120min (supposedly shown by the yellow arrow, but the yellow arrow just shows the main nucleus at 120min).

Right. Thanks for pointing this out. The arrow was slightly mispositioned. We have now moved it to point directly at the micronucleus or nuclear bleb (now Fig S2B).

S1B: what happens to the second nucleus in RNAi Ankle2? Does it get out of focus or collapses together with the only nucleus visible? Also, why does Lamin remain largely intranuclear in the control, rather than staining the nuclear rim? Is it just a problem of z-projection?

The second nucleus in the RNAi Ankle2 cell goes out of focus at 60 min but reappears in focus at 120 (on the right). In the control cell, Lamin appears more intranuclear due to the z-projection of the spheroid nuclei. Note that this is now Fig S2B.

S3: would it be possible to add a panel showing lamin RNAi and the levels of cDcp1?

Yes. We have now added the requested images (now in Fig S4A, right).

Introduction:

1) I suggest introducing micronuclei and the mechanisms that lead their formation (most often, mitotic errors). I also recommend including reference to the pivotal work from Kato and Sandberg (PMID 5635016) and the Pellman lab (PMID 22258507) and explaining why DNA isolation in micronuclei is associated with formation of DNA damage (e.g. incomplete replication and exposure of DNA to the cytoplasm). In addition, I believe the paragraph does not flow well with the surrounding text. Could the authors consider a different placement?

We have re-worked the text about micronuclei in the introduction to incorporate several of the elements suggested by the reviewer. The paragraph about micronuclei has also been moved up and adjustments have been made to the other paragraphs to improve the flow.

2) Regarding whether micronuclei can trigger apoptosis, please note that most evidence supporting apoptosis after micronucleation relies on treatment with pleiotropic, cytotoxic drugs. I am not sure whether micronucleation per se was reported to function as an apoptotic stimulus, and whether a clear-cut difference was made between the apoptotic potential of micronucleation vs aneuploidization.

Yes, we agree. This is why we are being careful in our introduction, by writing that it has been suggested that micronuclei can trigger apoptosis.

3) I advise against the use of the acronym NR (for Nuclear Reassembly). While other acronyms are common (like NE for Nuclear Envelope), this is unusual and does not help readability of the manuscript

We have introduced this acronym because we use the term Nuclear Reassembly (NR) 64 times in our manuscript. The mechanisms we have studied here do not impact only Nuclear Envelope Reassembly/Reformation (NER, previously used in other publications), but also the cohesion of the chromatin mass in the reassembling nucleus. We feel that the use of the acronym NR is justified and helps keep the text briefer.

Results:

1) Definition of D-mel abbreviation is missing (should be indicated when first mentioned)

D-Mel is not an abbreviation in this case but the name of the cell line (also known as d.mel-2, as indicated in the Materials and Methods section). We have now specified the name d.mel-2 in parentheses after the first mention of D-Mel in the Results section.

2) "Depletion of Ankle2, BAF or Lamin also caused delocalization of Nuclear Pore Complex (NPC) proteins and Otefin (an inner nuclear membrane protein with a LEM domain that interacts with BAF) from the NE (Fig S3D)" - I believe the authors refer to Fig S3C. I find it very surprising that BAF knock down causes a milder phenotype than Ankle2, if Ankle2 effects are mostly due to its role in BAF dephosphorylation. Is this due to the use of a weaker RNAi line?

We have corrected the figure number; thanks (now Fig S4C). The images shown are meant to provide a merely qualitative idea (not quantitative) of the presence or absence of phenotypes. The level of defects is variable, even within a single wing disc. Nevertheless, the phenotype may be stronger after Ankle2 RNAi than after BAF RNAi if BAF is harder to deplete (because more abundant or stable) or if a smaller fraction of BAF is sufficient for its function. In cells in culture, the percentages of cells with abnormal nuclei is lower for BAF RNAi than Ankle2 RNAi after 4 days, but similar between the two after 7 days (Fig 2C).

3) Although it is clear why overexpression of GFP-BAF3A rescues Ankle2 depletion, it is less clear why any rescue should occur by overexpressing GFP-BAFWT. Could the authors elaborate? Do they expect a fraction of the WT protein to escape phosphorylation?

Yes, this is how we tend to interpret this result. Protein phosphorylation or dephosphorylation of a given substrate is rarely complete in cells. Moreover, in this case Ankle2 depletion is likely partial (the weaker RNAi line is used in this experiment), which would allow partial dephosphorylation of overexpressed GFP-BAF WT. We have added this sentence:

"The fact that some rescue is observed suggests that a pool of overexpressed GFP-BAF^{WT} remains unphosphorylated even upon partial depletion of Ankle2."

4) Figure S4D: what is the explanation for lack of statistical significance in the TUNEL assay between control and BAF RNAi?

Although a difference is expected, and is suggested by the observed trend, the p-value is below the standard significant threshold for this particular comparison because of the intrinsic variability of the experiment and because the sample numbers are relatively low.

Rev. 2:
In this manuscript, Li et al. dissect the molecular mechanisms promoting nuclear envelope reformation (NER) during mitotic exit and investigate the cellular and physiological consequences of nuclear reformation defects on tissue development in Drosophila melanogaster.
In the first part of the manuscript, they provide evidence that Anlke2/Lem4 promotes BAF dephosphorylation during mitotic exit to induce NER. Inactivation of Ankle2/Lem4 prevents BAF recruitment to the chromosomes, and leads to major NER defects. The inactivation of BAF itself recapitulates this phenotype. These observations are consistent with previous observations in mammalian cells and in C. elegans embryos.
In the second part, using the Drosophila wing as a model system, they evaluate the physiological consequences of nuclear reformation defects on tissue development. They nicely show that NER defects lead to the development of smaller wings, and that this phenotype can be enhanced or suppressed using mutations aggravating or suppressing (respectively) NER defects. They further show that NER defects trigger an apoptotic response, which is required for the wing tissue to recover from the NER- defect-induced damage. Activation of apoptosis in response to NER defects involves the activation of the DNA damage response kinase Chk2, but is independent of p53 activation. Nevertheless, in response to NER defects, p53 promotes a cell cycle arrest in G1 via the ubiquitin-mediated degradation of Cyclin E. Both the apoptotic response and the cell cycle arrest are beneficial to wing tissue development.

Overall, this is an interesting manuscript that nicely links cellular defects to tissue development. However, I found several parts confusing and needing clarification.

Specific points:

- The Drosophila genome possesses two genes encoding lamins, Lamin C and lamin Dm0 but it is unclear which lamin the authors are monitoring. When they do lamin RNAi, what does it mean? Do they inactivate only one lamin or both? This information is critical to understand the results. Furthermore, the authors monitor GFP-lamin localization, is this fusion protein fully functional?

Lamin is the preferred name of Dm0, according to Flybase. This is the only lamin we are referring to in all our experiments. It is the most ubiquitously expressed lamin. GFP-Lamin (GFP-Dm0) is used merely to follow Lamin localization during nuclear envelope reassembly in our experiments. We have not tested if this fusion protein is fully functional in a genetic rescue experiment. Nevertheless, the defects of GFP-Lamin localization after Ankle2 or BAF RNAi mirror the defects we observed by staining for endogenous Lamin.

- Page 8, at the beginning of the paragraph "Nuclear reassembly defects trigger apoptosis": the authors should state more clearly that they are monitoring apoptosis in the fly wings. A non-

Drosophila reader would not really know what tissue is being looked at. This information eventually appears only on line 12.

We have clarified it in the text.

- The statistical analyses are questionable and some information is missing (n= sample size and N=number of independent experiments performed). On several graphs, the authors add a p value <0.0001 (****) but just by looking at the data points between conditions, one can wonder how an unpaired t-test can give such a low p value. For instance, Fig2B where the authors compare the effect of lamin-, BAF- or Ankl2-RNAi on nuclear solidity to the control. In all cases, comparison to the control gives a p value <0.0001, and yet Ankle2 RNAi appears to have a much stronger effect. The same comment applies to Figures 3B, 7A, S1C. Maybe the authors could double-check their statistical analysis.

We have now specified the sample size and number of experiments where this information was missing. We have double-checked our statistical tests and they are correct. In many cases (as in Fig 2B), the p value is very low despite a small difference, because the numbers of values in each condition are very high. In other case (as in Fig 3B), the p value is very low despite a small difference, because the variance of the values is very low. As we verified our data and figures, we noticed that not all data points were displayed in Figures 1C, 1D and 2B. We have now fixed this problem.

- Please use the same nomenclature for the RNAi between the figures and the text.

We have done these clarifications.

- All the interactions in Figure 7 are entirely based on genetic interactions, which are nicely designed and conducted, but confirming these interactions using molecular markers would strengthen the conclusions. For instance, demonstrating that introduction of an ago mutation directly causes Cyclin E accumulation in their system would consolidate the conclusions. Likewise, presenting molecular evidence directly showing that p53 is activated in response to NER defects would be desirable.

We understand the suggestion and agree that such results would be good. However, we searched hard for a reliable Cyclin E antibody but could not find one. The antibody that everyone previously used in the field was discontinued. Regarding p53, we emphasize that we do not conclude that p53 is activated upon nuclear reassembly defects, but that it is required. We did not find an antibody that would specifically recognize activated p53. Please note that we are being conservative in our interpretations of our results and in the conclusions we draw regarding the precise molecular mechanism of the p53-dependent response to nuclear reassembly defects. Please also note our response to Reviewer 1's point 2.

Figure 1:
Figure 1B: RNAi Ankle 2 and RNAi NT panels are inverted. Also, the authors should write "control" instead of "NT" because NT is not self-explanatory.

We have corrected the inverted labeling. We have also replaced RNAi NT with RNAi Control in all figures, and specified in the legend of Fig 1 that "RNAi Control = Non-target dsRNA against bacterial kanamycin resistance gene".

Figure 1B and Figure 1D: the authors mentioned that they quantified BAF levels at the NE but they did not use any nuclear envelope marker for the quantification! How did they quantify the NE levels of BAF in these conditions? This information is not provided in the Materials and Methods section.

We have measured the fluorescence at the nuclear periphery, ie at the edges of the DNA staining. This is now explained in the Materials and Methods section.

Figure 1B: Why is there less FLAG-BAF signal in the Ankle RNAi compared to the RNAi Ctrl? This panel is confusing when we compare it to the first panel presented Figure 1E (-43 min). The authors should clarify this point.

To us, it does not seem like the total Flag-BAF signal is much lower in the Ankle2 RNAi cell than in the Ctrl RNAi cell. In any case, the total signal varies between cells in this cell line which is not clonal. In Figure 1E, we show an Ankle2 RNAi cell that starts with little or no nuclear phenotype and develops the phenotype as it exits from mitosis. This is what we typically see. After transfection with dsRNA, cells need to go through mitosis to develop the phenotype as they attempt to reassemble their nuclei in the absence of Ankle2. Once their nuclei are strongly damaged (as in Fig 1B), they do not divide.

Figure 1E: The authors should present pics taken with the same timing intervals and maybe also include an extra panel, to show that even after 113 min, BAF is still not recruited to the NE after Ankle2 inactivation. Some points are missing in the image (the highest GFP-BAF level is at 7 min), which are required for the interpretation of the graph (GFP-BAF recruitment to reassembling nuclei).

We have added several microscopy images to the figure as suggested.

Figure 1E: Add a panel showing BAF 3A localization during mitosis.

We have now added new data showing the localization in GFP-BAF^{3A} throughout mitosis (New Fig S1). As we showed previously (Mehsen et al, 2018), GFP-BAF^{3A} remains localized to chromosomes during mitosis, unlike GFP-BAF^{WT}. In addition, our new results show that expression of GFP-BAF^{3A} rescues defects in Lamin recruitment to reassembling nuclei upon depletion of Ankle2, consistent with our model (New Fig S1B).

Figure 1E: As mentioned above, at -44.3 min (interphase), GFP-BAF presents the exact same localization in the NT and RNAi treated cells, whereas in Figure 1B in the Ankle RNAi the GFP-BAF is not in the NE! This is confusing and should be clarified.

Please see our response above. After transfection with dsRNA, cells need to go through mitosis to develop the phenotype as they attempt to reassemble their nuclei in the absence of Ankle2. We have now clarified it in the text.

Figure 1F: As the authors mentioned previous work performed on C. elegans BAF. It would be useful to include it in the alignment.

We have now added the *C. elegans* sequence to our alignment.

Figure 1G: This western blot would be more convincing if the authors include a loading control and quantification.

We have now repeated the experiment, including a loading control (α-Tubulin) and quantifications. The result is consistent with our previous results but the suggested improvements to the analysis make it more conclusive. The new data is in Fig 1H.

Figure 2: Again, please mention which lamin is monitored in panel A. In Panel B, explain in more detail the quantification (define "solidity"). In addition, this graph should start at "0". Panel D: Explain what is considered as "abnormal Lamin", and how was it quantified?

In all experiment, the lamin monitored is Lamin (Dm0, now specified in the text). Lamin is its official name according to Flybase. The only other lamin in *Drosophila* is called Lamin C, which is not ubiquitously expressed. We have now defined "nuclear solidity" and "abnormal Lamin" in the Materials and Methods where quantification methods are described. We have changed Fig 2B to make the axis start at 0. We have clarified how abnormal Lamin was quantified in the legend and in the Materials and Methods.

Figure 4, panel B: There are clearly more Dcp-1 dots in Ankle2 RNAi compared to Lamin RNAi on the presented images but the graph in Figure 4C shows the opposite. This is confusing. Panel E: add Dcp-1/Tunel for the images presented on top and Tunel/DNA for the images at the bottom.

We have replaced the images in Fig 4B with more representative images. We have fixed the labeling in Fig 4E.

Figure S3B: Compare TioOE with BAF RNAi #1 (40D) because this is the real control and not the No RNAi. Verify the statistical analysis p value.

We have added the missing statistical analysis (now Fig S4B).

Figure S3C: Which antibody was used to detect the NPCs?

The antibody used is already specified in the Materials and Methods section (Ab24609, Abcam).

We thank the reviewers once again for their constructive input.

Vincent Archambault
